# Predicting 12-month functional outcome in Guillain-Barré syndrome by combining acute-phase clinical data and traditional Chinese medicine syndrome features: a retrospective machine learning study

**DOI:** 10.3389/fneur.2026.1859051

**Published:** 2026-07-29

**Authors:** Peng Hu, Jingming Liu, Yan Tang, Jiantao Huang, Xinyi Chen, Chang Liu, Yuxing Wang

**Affiliations:** 1 Department of Encephalopathy, Changde First Hospital of Traditional Chinese Medicine, Changde, Hunan, China; 2 Department of Medical Affairs, Changde First Hospital of Traditional Chinese Medicine, Changde, Hunan, China

**Keywords:** 12-month outcome, acute-phase prediction, calibration, deep learning, Guillain-Barré syndrome, machine learning, traditional Chinese medicine syndrome

## Abstract

**Background:**

Functional recovery after Guillain-Barré syndrome (GBS) varies substantially. Prediction models that combine routinely available acute-phase clinical information with traditional Chinese medicine (TCM) syndrome features have not been adequately reported or validated. This study developed an acute-phase dynamic prediction model for 12-month functional outcome in GBS and evaluated discrimination, calibration, and potential clinical utility.

**Methods:**

We retrospectively screened 601 patients admitted between January 2015 and December 2023; 525 met eligibility criteria and had 12-month outcome data. The prediction index time was the end of acute in-hospital assessment/discharge; no post-discharge follow-up variables were used as predictors. Candidate predictors included demographics, acute clinical severity, laboratory and electrophysiological findings, treatment-related acute-care variables, and standardized TCM syndrome elements. Poor outcome was defined as Hughes Functional Grading Scale (HFGS) score > = 3 at 12 months. Patients were stratified into a training cohort (*n* = 368) and a hold-out validation cohort (*n* = 157). Preprocessing, imputation, standardization, feature selection, and synthetic oversampling were performed within the training data only. Logistic regression, random forest, and a fully connected neural-network model were compared. Performance was assessed with 95% confidence intervals, calibration plots, Brier score, decision-curve analysis, and formal AUC comparisons.

**Results:**

Poor 12-month outcome occurred in 148 of 525 patients (28.2%). Mechanical ventilation, admission HFGS score, AMSAN subtype, qi deficiency, and blood stasis were independently associated with poor outcome. In the hold-out validation cohort, AUCs were 0.744 (95% CI 0.655–0.833) for logistic regression, 0.843 (95% CI 0.780–0.906) for random forest, and 0.887 (95% CI 0.831–0.943) for the neural network. The neural network had the lowest Brier score (0.123) and showed acceptable calibration (calibration intercept 0.03; slope 0.94). DeLong testing showed that the neural network outperformed logistic regression (*p* = 0.006) but was not statistically superior to random forest (*p* = 0.174). Decision-curve analysis suggested net benefit for the machine-learning models across clinically plausible thresholds.

**Conclusion:**

In this single-center retrospective cohort, acute-phase clinical variables and TCM syndrome elements were associated with 12-month functional outcome in GBS. The neural-network model had the highest numerical AUC and lowest Brier score, but it was not statistically superior to random forest; therefore, no superiority over random forest is claimed. External validation, comparison with established prognostic scores when required variables are available, and prospective implementation studies are required before clinical use.

## 1 Introduction

Guillain-Barré syndrome is an acute immune-mediated neuropathy that can progress rapidly from mild limb weakness to respiratory failure and prolonged dependency. Even in the era of immunotherapy and modern supportive care, recovery is uneven. Some patients regain independent ambulation relatively quickly, whereas others continue to experience marked disability many months after onset. This heterogeneity makes prognostic assessment clinically valuable for monitoring intensity, counseling, rehabilitation planning, and follow-up allocation (1–3).

Previous studies have linked outcome in GBS to age, neurological severity, respiratory involvement, autonomic dysfunction, and electrophysiological subtype, especially axonal forms of disease (4–8). However, the intended prediction time point is critical. Models using only admission variables can support early triage, whereas models that include nadir disability or mechanical ventilation during hospitalization are better understood as acute-phase dynamic or discharge-time prognostic tools. This distinction is necessary to avoid data leakage and to ensure that all predictors are available before model deployment.

In clinical settings where integrative medicine is routinely practiced, TCM syndrome differentiation represents an additional layer of patient characterization. Within the TCM framework, GBS is commonly discussed under the broader concept of Wei syndrome, and patterns such as qi deficiency, dampness accumulation, and blood stasis are recorded in routine practice. These variables may summarize clinically observed constitutional or recovery-related features, but their prognostic meaning should be interpreted as hypothesis-generating rather than causal.

Machine learning methods offer a way to integrate multidimensional information without assuming simple linear relationships. Algorithms such as random forest and neural networks can model nonlinear relations and interactions, but reporting must include transparent feature definitions, preprocessing, training details, calibration, and uncertainty estimates (9–17). Against this background, we developed and internally validated prediction models for poor 12-month functional outcome in GBS using acute-phase clinical data and TCM syndrome features. We compared logistic regression, random forest, and a neural-network model and evaluated discrimination, calibration, and decision-curve-based clinical utility.

## 2 Materials and methods

### 2.1 Study design and patient population

**Figure 1:**
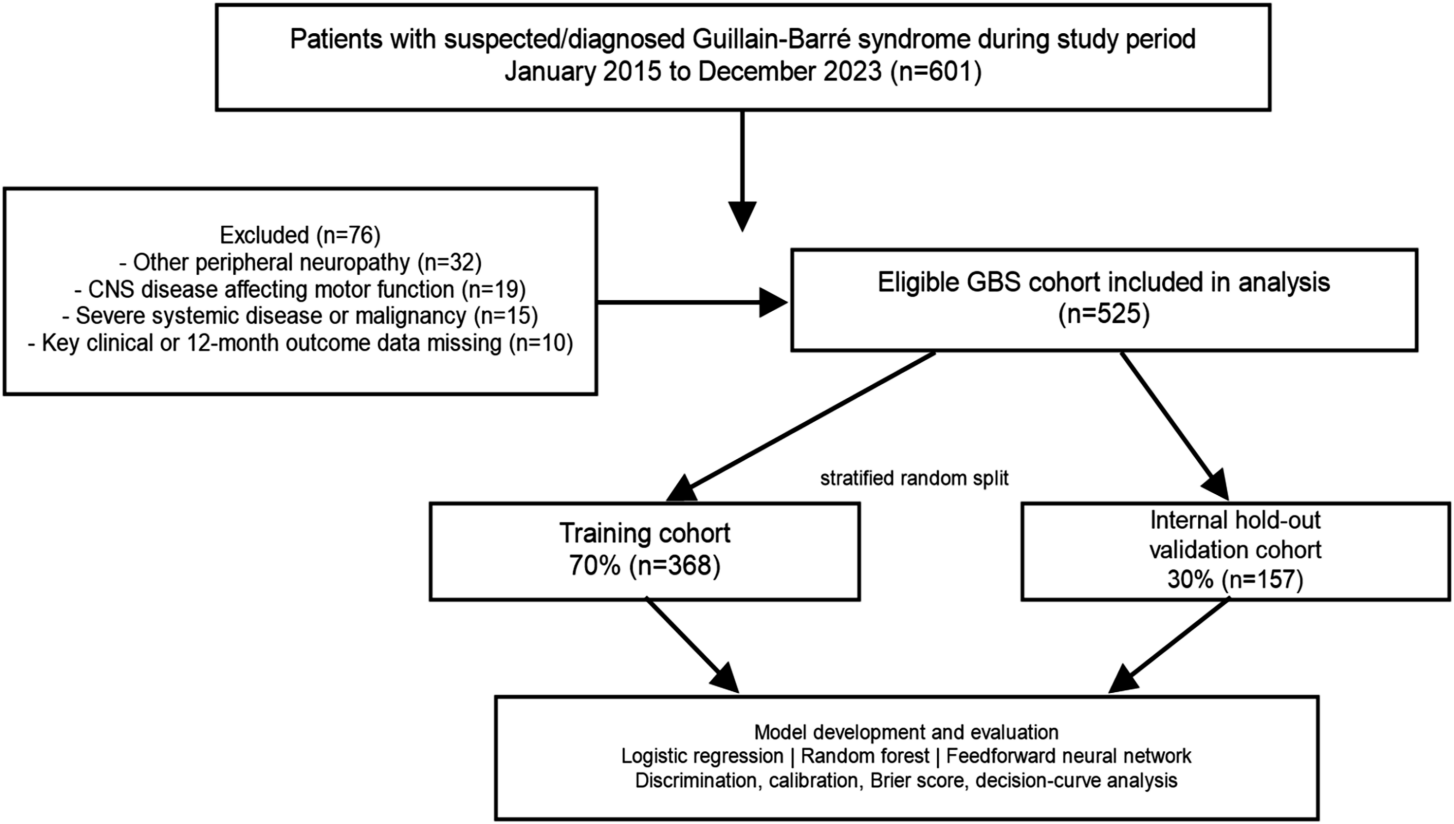
Flowchart of cohort assembly, exclusion criteria, data allocation, and model evaluation. A total of 601 patients were screened, 525 were included, and eligible cases were randomly assigned to the training cohort (70%) and hold-out validation cohort (30%).

This retrospective cohort study screened consecutive patients with suspected or diagnosed GBS who were admitted to the Department of Neurology at Changde First Hospital of traditional Chinese medicine between January 2015 and December 2023. A total of 601 patients were screened. After applying exclusion criteria, 525 patients were included in the final analysis (Figure 1).

The diagnosis of Guillain-Barré syndrome (GBS) was established by review of clinical presentation, neurological examination, cerebrospinal fluid findings when available, and nerve-conduction/electromyography findings, using Brighton Collaboration case-definition levels 1–3 (18). In brief, eligible patients had bilateral flaccid limb weakness, decreased or absent tendon reflexes, a monophasic disease course with interval from onset to nadir between 12 h and 28 days, no better alternative diagnosis, and supportive cerebrospinal fluid or electrophysiological evidence when available. Electrophysiological subtypes were classified according to the criteria of Hadden et al. (19). Acute inflammatory demyelinating polyneuropathy (AIDP) was defined by demyelinating features such as prolonged distal motor latency, slowed motor conduction velocity, prolonged or absent F waves, conduction block, or temporal dispersion in the required number of nerves. Acute motor axonal neuropathy (AMAN) was defined by motor axonal involvement without demyelinating criteria and with preserved sensory nerve action potentials, whereas acute motor-sensory axonal neuropathy (AMSAN) was defined by both motor and sensory axonal involvement. In this retrospective dataset, subtype coding was limited to AIDP, AMAN, and AMSAN; cases with equivocal, incomplete, or unclassifiable electrophysiological data were treated as missing key diagnostic/subtype information and were not included in the final analytic cohort.

Patients were eligible if they were at least 18 years old, had complete baseline clinical information, underwent electrophysiological testing, received standardized TCM syndrome differentiation during hospitalization, and had 12-month HFGS outcome data. Patients were excluded if another cause of peripheral neuropathy was identified, if a central nervous system disorder clearly affected motor function, if severe systemic disease or malignancy was expected to dominate prognosis, or if key clinical variables or 12-month outcome data were missing.

### 2.2 Prediction time point and leakage prevention

The intended use of the model was risk stratification at the end of acute in-hospital assessment/discharge to guide rehabilitation intensity, follow-up scheduling, and counseling. Therefore, the prediction index time was defined as discharge from acute hospitalization after completion of routine acute-phase assessments. Variables obtained after discharge, including outpatient follow-up findings and the 12-month HFGS outcome, were not used as predictors.

Follow-up information was used only to ascertain the primary endpoint. Variables that could occur during the acute hospitalization, such as nadir HFGS score and mechanical ventilation, were retained because they were known before discharge. Accordingly, the model should not be interpreted as an admission-only early prediction model; it is an acute-phase dynamic/discharge-time prognostic model.

To prevent analytic leakage, all imputation parameters, standardization parameters, feature-selection decisions, threshold selection, and resampling procedures were fitted using training data only. The hold-out validation cohort was not used during preprocessing, tuning, model selection, or threshold optimization.

### 2.3 Variables and endpoint definition

Candidate predictors extracted from the electronic medical record included age, sex, body mass index, antecedent infection, interval from symptom onset to admission, cranial nerve involvement, autonomic dysfunction, mechanical ventilation during acute hospitalization, HFGS score at admission, HFGS score at nadir, cerebrospinal fluid protein, serum albumin, C-reactive protein, electrophysiological subtype, acute immunotherapy, early rehabilitation referral, and TCM syndrome elements. The complete candidate feature set and preprocessing plan are shown in Supplementary Table S1.

The primary endpoint was poor functional outcome at 12 months after symptom onset, defined as HFGS score > = 3. Patients with HFGS score 0–2 were categorized as having favorable outcome. To address the limited granularity of this dichotomized endpoint, we also performed exploratory sensitivity analyses using the ordinal 12-month HFGS score and the change in HFGS score from admission to 12 months.

The 12-month HFGS outcome was calculated from symptom onset. Whenever possible, HFGS was assessed during outpatient follow-up; when in-person assessment was not feasible, trained clinicians conducted a structured telephone interview using standardized questions on independent walking, need for walking aid or assistance, bed/chair confinement, ventilatory support, and vital status. Death before the 12-month assessment was coded as HFGS grade 6 and included in the poor-outcome category. Outcome assessors were not involved in model development, hyperparameter tuning, or threshold selection and were blinded to model predictions. Because this was a retrospective clinical cohort, complete blinding to all acute clinical information could not be guaranteed. Patients lost to follow-up or with unverifiable 12-month outcome status were excluded before cohort splitting and are included in the flowchart category of missing key clinical variables or 12-month outcome data.

### 2.4 Standardization of TCM syndrome differentiation

TCM syndrome elements were recorded using a pre-specified hospital syndrome-differentiation form based on the WHO International Standard Terminologies on Traditional Medicine in the Western Pacific Region and Chinese national diagnostic standards/guidance for TCM disease and syndrome classification, including Wei syndrome and peripheral neuropathy (20–22). Elements included qi deficiency, dampness accumulation, blood stasis, phlegm turbidity, yin deficiency, yang deficiency, and heat toxin. Each element was coded as present or absent, and multiple syndrome elements could coexist in the same patient when criteria for more than one element were met.

Operational definitions were specified before data extraction. Qi deficiency required fatigue or reduced strength with at least one supportive sign such as shortness of breath, spontaneous sweating, pale tongue, or weak pulse. Dampness accumulation required limb heaviness, numbness, edema or sticky stool with greasy tongue coating or slippery pulse. Blood stasis required fixed pain or numbness, dark or purplish tongue, petechiae/ecchymosis, or choppy pulse. Phlegm turbidity required heaviness, chest oppression, copious sputum or turbid coating with slippery pulse. Yin deficiency required dry mouth, night sweating or heat sensation, red tongue with scant coating, or thready rapid pulse. Yang deficiency required cold intolerance, cold limbs, pale swollen tongue, or deep weak pulse. Heat toxin required fever, sore throat/thirst, inflammatory manifestations, red tongue with yellow coating, or rapid pulse.

Syndrome differentiation was performed within 48 h of admission by two attending clinicians trained in both neurology and TCM. Before data extraction, evaluators received standardized training on operational definitions, tongue and pulse descriptors, symptom documentation, and coding rules. Evaluators were not blinded to the clinical manifestations required for syndrome differentiation, but they were blinded to the 12-month functional outcome and did not participate in outcome assessment or model training. Disagreements were resolved by consensus with a senior TCM neurologist. Inter-rater reliability was assessed in a randomly selected subset of 80 records; agreement was substantial for the most frequent elements: qi deficiency kappa = 0.78, dampness accumulation kappa = 0.72, blood stasis kappa = 0.75, and overall element-level kappa = 0.74.

### 2.5 Model development

The full cohort was divided into a stratified random training cohort (70%, *n* = 368) and an internal hold-out validation cohort (30%, *n* = 157) using a fixed random seed. Continuous variables were standardized with *z*-score scaling, and categorical predictors were one-hot encoded. Records missing key eligibility or endpoint information, including unavailable 12-month HFGS outcome or unavailable/unclassifiable electrophysiological subtype, were excluded before cohort splitting as specified in the flowchart. In the analytic cohort, item-level missingness for candidate predictors was low (0–5.7%; Supplementary Table S5).

Missing data were handled within the training data to avoid leakage. For logistic regression, chained-equation multiple imputation with 20 imputed datasets was used for variables with item-level missingness, and regression estimates were pooled across imputations according to Rubin’s rules. For random forest and neural-network models, imputation was implemented inside the machine-learning pipeline using training-fold median imputation for continuous variables and most-frequent-category imputation for binary or categorical variables. During cross-validation, imputation models were refitted within each training fold. The hold-out validation cohort was transformed only with imputation and scaling parameters learned from the training data.

To address class imbalance, the Synthetic Minority Oversampling Technique was applied only within the training data and, during cross-validation, only within the training fold. It was never applied to the hold-out validation cohort. Model tuning and internal optimization were performed with five-fold cross-validation in the training cohort.

Feature selection was performed within the training data before final model fitting. Candidate variables were retained when they were prespecified as clinically important or showed a univariable association with poor outcome at *p* < 0.10. The final selected feature set contained age > = 60 years, time from symptom onset to admission, autonomic dysfunction, mechanical ventilation, admission HFGS score, nadir HFGS score, cerebrospinal fluid protein, C-reactive protein, electrophysiological subtype, qi deficiency, and blood stasis. Electrophysiological subtype was encoded using two dummy variables (AMAN and AMSAN, with AIDP as the reference), producing 12 encoded input features for the extended models and 10 encoded input features for the biomedical-only reference models. The same selected feature sets and validation patients were used for logistic regression, random forest, and neural-network analyses (Supplementary Table S4).

Logistic regression used L2 regularization, with the regularization parameter selected by grid search. The random forest classifier used 500 trees; candidate values for maximum depth, minimum samples per leaf, and maximum features were tuned by grid search. The final random forest used maximum depth = 6, minimum samples per leaf = 5, and max_features = sqrt.

The deep learning model was a compact fully connected feedforward neural network. The input layer corresponded to the final encoded feature vector. Hidden layers contained 64, 32, and 16 neurons with rectified linear activation. Dropout rates were 0.30 after the first hidden layer and 0.20 after the second hidden layer. L2 weight decay was 1e-4. The output layer used sigmoid activation. With 12 encoded inputs in the extended model, the neural network contained 3,457 trainable parameters (832 in the input-to-first-hidden layer, 2,080 and 528 in the next hidden layers, and 17 in the output layer). The biomedical-only neural network contained 3,329 trainable parameters. The model was trained with binary cross-entropy loss and the Adam optimizer (learning rate = 0.001), batch size = 32, maximum 300 epochs, and early stopping with patience = 25 based on validation-fold loss. The classification threshold was selected in the training cohort by maximizing the Youden index and then fixed before internal hold-out validation. Given the modest number of poor-outcome events, the neural-network analysis was treated as exploratory and protected against overfitting through a small architecture, regularization, dropout, early stopping, cross-validation, and complete separation of the internal validation cohort from all training and tuning steps.

To directly assess whether TCM syndrome features added prognostic information beyond conventional biomedical predictors, we specified paired reference and extended models. The biomedical reference models contained the same selected conventional predictors but excluded qi deficiency and blood stasis. The extended models added these two TCM syndrome elements while keeping the modeling algorithm, preprocessing pipeline, resampling strategy, tuning procedure, and validation patients identical. Incremental performance was assessed by change in AUC, paired DeLong tests, Brier score, calibration, and decision-curve analysis in the internal hold-out validation cohort.

Analyses were performed in Python 3.10.13 using pandas 2.2.2, NumPy 1.26.4, scikit-learn 1.4.2, imbalanced-learn 0.12.3, TensorFlow 2.16.1/Keras 3.3.3, SciPy 1.13.1, and Matplotlib 3.8.4.

### 2.6 Model assessment and statistical analysis

Model performance was assessed in the hold-out validation cohort using accuracy, sensitivity, specificity, positive predictive value, negative predictive value, F1 score for the poor-outcome class, and the area under the receiver operating characteristic curve (AUC). Ninety-five percent confidence intervals for classification metrics were calculated with Wilson intervals, and AUC confidence intervals were estimated with 1,000 bootstrap resamples.

Calibration was evaluated with calibration plots, calibration intercept, calibration slope, and Brier score. Decision-curve analysis was used to estimate net benefit across threshold probabilities (29). Pairwise AUC comparisons were performed with DeLong tests. All validation analyses in this study were internal. To strengthen internal validation in the absence of an external cohort, we performed 1,000 bootstrap optimism correction, repeated five-fold cross-validation, and a temporal validation sensitivity analysis in which earlier cases were used for training and later cases for testing.

We considered comparison with established GBS prognostic scores, including the modified Erasmus GBS Outcome Score and the Erasmus GBS Respiratory Insufficiency Score (23–25). A formal head-to-head comparison was not performed because several required score variables, particularly Medical Research Council sum score, preceding diarrhea, and the exact 2-week GBS disability score used in these scores, were not consistently available in the retrospective dataset. Therefore, the present models are not interpreted as superior to established validated prognostic tools.

Continuous variables are reported as mean ± standard deviation or median with interquartile range according to distribution, and categorical variables as counts and percentages. Group comparisons used Student’s t test, the Mann–Whitney *U* test, chi-square test, or Fisher’s exact test as appropriate. A two-sided *p*-value <0.05 was considered statistically significant.

## 3 Results

### 3.1 Cohort profile and comparability of training and validation sets

Among 601 screened patients, 76 were excluded and 525 were included in the final cohort. Exclusion reasons were other causes of peripheral neuropathy (*n* = 32), central nervous system diseases affecting motor function (*n* = 19), severe systemic disease or malignant tumor expected to influence functional outcome (*n* = 15), and missing key clinical variables or 12-month outcome data (*n* = 10). The cohort was stratified into 368 patients in the training set and 157 in the hold-out validation set (Figure 1).

Poor 12-month outcome occurred in 104 patients (28.3%) in the training cohort and 44 patients (28.0%) in the validation cohort. The two cohorts were balanced in demographic characteristics, major clinical manifestations, electrophysiological subtype, laboratory values, and TCM syndrome elements (Table 1). The most frequent syndrome elements were qi deficiency, dampness accumulation, and blood stasis.

**Table 1:** Characteristics of the training and validation cohorts.

Variable	Training cohort (*n* = 368)	Validation cohort (*n* = 157)	*p*-value
Age (years)	51.6 ± 15.2	52.1 ± 14.9	0.72
Male sex, *n* (%)	224 (60.9)	96 (61.1)	0.97
BMI (kg/m^2^)	23.7 ± 3.4	23.5 ± 3.3	0.54
Antecedent infection, *n* (%)	196 (53.3)	81 (51.6)	0.71
Time to admission (days)	5.8 ± 2.9	6.1 ± 3.1	0.32
Cranial nerve involvement, *n* (%)	114 (31.0)	47 (29.9)	0.79
Autonomic dysfunction, *n* (%)	82 (22.3)	33 (21.0)	0.73
Mechanical ventilation, *n* (%)	41 (11.1)	18 (11.5)	0.90
HFGS score at admission	3.6 ± 0.9	3.7 ± 1.0	0.44
HFGS score at nadir	4.2 ± 0.8	4.1 ± 0.9	0.38
CSF protein (g/L)	1.21 ± 0.53	1.18 ± 0.49	0.61
Serum albumin (g/L)	36.8 ± 4.7	37.1 ± 4.5	0.48
C-reactive protein (mg/L)	10.5 ± 7.6	10.1 ± 7.4	0.59
Electrophysiological subtype			0.66
AIDP	189 (51.4)	83 (52.9)	
AMAN	114 (31.0)	46 (29.3)	
AMSAN	65 (17.7)	28 (17.8)	
TCM syndrome elements			
Qi deficiency	202 (54.9)	86 (54.8)	0.99
Dampness accumulation	176 (47.8)	72 (45.9)	0.69
Blood stasis	149 (40.5)	64 (40.8)	0.95
Phlegm turbidity	128 (34.8)	52 (33.1)	0.70
Yin deficiency	92 (25.0)	37 (23.6)	0.73
Yang deficiency	61 (16.6)	27 (17.2)	0.86
Heat toxin	52 (14.1)	21 (13.4)	0.82

GBS, Guillain-Barré syndrome; BMI, body mass index; CSF, cerebrospinal fluid; CRP, C-reactive protein; AIDP, acute inflammatory demyelinating polyneuropathy; AMAN, acute motor axonal neuropathy; AMSAN, acute motor-sensory axonal neuropathy; TCM, traditional Chinese medicine.

### 3.2 Variables associated with poor 12-month functional outcome

Univariate analysis identified several variables related to poor outcome, including age > = 60 years, delayed admission, autonomic dysfunction, mechanical ventilation, higher admission HFGS score, higher nadir HFGS score, elevated cerebrospinal fluid protein, higher C-reactive protein, AMSAN subtype, qi deficiency, and blood stasis (Table 2).

**Table 2:** Univariable and multivariable analyses of factors associated with poor 12-month outcome.

Variable	Univariate OR (95% CI)	*p*-value	Multivariable OR (95% CI)	*p*-value
Age > = 60 years	1.74 (1.21–2.48)	0.003	1.32 (0.89–1.95)	0.160
Male sex	1.12 (0.78–1.60)	0.540	—	—
BMI	1.03 (0.97–1.09)	0.310	—	—
Antecedent infection	1.21 (0.84–1.74)	0.300	—	—
Time to admission	1.05 (0.99–1.12)	0.087	1.03 (0.97–1.10)	0.291
Cranial nerve involvement	1.33 (0.91–1.95)	0.140	—	—
Autonomic dysfunction	1.64 (1.09–2.47)	0.018	1.29 (0.82–2.04)	0.270
Mechanical ventilation	2.68 (1.63–4.41)	<0.001	2.21 (1.29–3.78)	0.004
HFGS score at admission	1.52 (1.21–1.91)	<0.001	1.41 (1.10–1.80)	0.007
HFGS score at nadir	1.35 (1.07–1.71)	0.012	1.18 (0.91–1.52)	0.210
CSF protein	1.39 (1.02–1.89)	0.035	1.18 (0.84–1.66)	0.330
Serum albumin	0.96 (0.92–1.01)	0.104	—	—
CRP	1.07 (0.99–1.15)	0.080	1.04 (0.96–1.13)	0.322
AIDP	Reference	—	Reference	—
AMAN	1.29 (0.84–1.97)	0.240	1.18 (0.75–1.86)	0.470
AMSAN	1.71 (1.10–2.65)	0.016	1.67 (1.03–2.71)	0.037
Qi deficiency	1.48 (1.01–2.18)	0.044	1.52 (1.01–2.29)	0.043
Dampness accumulation	1.27 (0.88–1.82)	0.200	—	—
Blood stasis	1.56 (1.06–2.29)	0.024	1.61 (1.05–2.46)	0.028
Phlegm turbidity	1.19 (0.80–1.77)	0.390	—	—
Yin deficiency	1.14 (0.72–1.81)	0.570	—	—

OR, odds ratio; CI, confidence interval; BMI, body mass index; CSF, cerebrospinal fluid; CRP, C-reactive protein; AIDP, acute inflammatory demyelinating polyneuropathy; AMAN, acute motor axonal neuropathy; AMSAN, acute motor-sensory axonal neuropathy; HFGS, Hughes Functional Grading Scale.

In the multivariable model, five factors remained independently associated with poor prognosis: mechanical ventilation (OR 2.21, 95% CI 1.29–3.78, *p* = 0.004), higher admission HFGS score (OR 1.41, 95% CI 1.10–1.80, *p* = 0.007), AMSAN subtype (OR 1.67, 95% CI 1.03–2.71, *p* = 0.037), qi deficiency (OR 1.52, 95% CI 1.01–2.29, *p* = 0.043), and blood stasis (OR 1.61, 95% CI 1.05–2.46, *p* = 0.028). These associations should be interpreted as prognostic rather than causal.

### 3.3 Comparative model performance

**Figure 2:**
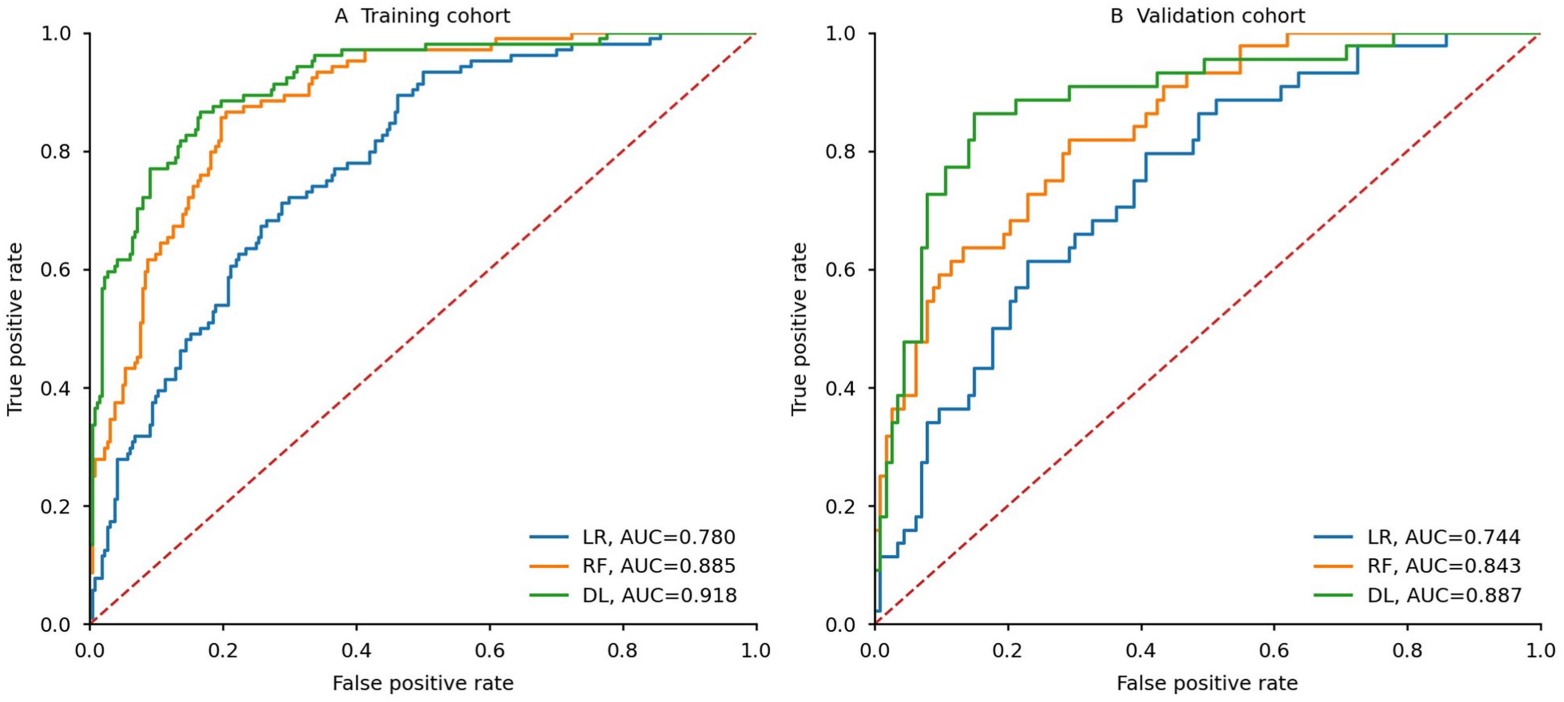
Receiver operating characteristic curves for logistic regression, random forest, and deep learning models in the training cohort **(A)** and validation cohort **(B)**. AUC values were harmonized with Table 3.

Performance values were checked and harmonized across text, tables, and figures (Table 3 and Figure 2). In the internal hold-out validation cohort, logistic regression achieved an AUC of 0.744 (95% CI 0.655–0.833), random forest achieved an AUC of 0.843 (95% CI 0.780–0.906), and the neural-network model achieved an AUC of 0.887 (95% CI 0.831–0.943).

**Table 3:** Discrimination and classification performance of the competing prediction models.

Model	Dataset	Accuracy (95% CI)	Sensitivity (95% CI)	Specificity (95% CI)	F1 score	AUC (95% CI)
Logistic regression	Training	0.78 (0.73–0.82)	0.70 (0.61–0.78)	0.81 (0.76–0.85)	0.64	0.780 (0.725–0.833)
Logistic regression	Validation	0.75 (0.68–0.81)	0.68 (0.53–0.80)	0.78 (0.69–0.85)	0.61	0.744 (0.655–0.833)
Random forest	Training	0.84 (0.80–0.87)	0.79 (0.70–0.86)	0.86 (0.81–0.90)	0.74	0.885 (0.846–0.923)
Random forest	Validation	0.81 (0.74–0.86)	0.75 (0.61–0.85)	0.83 (0.75–0.89)	0.69	0.843 (0.780–0.906)
Deep learning	Training	0.88 (0.84–0.91)	0.86 (0.78–0.91)	0.89 (0.85–0.92)	0.80	0.918 (0.887–0.949)
Deep learning	Validation	0.86 (0.80–0.91)	0.84 (0.71–0.92)	0.87 (0.79–0.92)	0.77	0.887 (0.831–0.943)

F1 score is reported for the poor-outcome class.

The neural network showed the highest numerical discrimination and classification performance, with accuracy 0.86, sensitivity 0.84, specificity 0.87, and F1 score 0.77 for the poor-outcome class in the internal hold-out validation cohort. However, formal comparison showed that the neural network significantly outperformed logistic regression (DeLong *p* = 0.006) but was not statistically superior to random forest (DeLong *p* = 0.174). Therefore, claims of model superiority were softened.

In the incremental-value analysis, the biomedical reference model and the extended model including TCM syndrome elements were compared using identical internal validation patients (Supplementary Table S3). In this analysis, adding TCM syndrome elements produced small-to-moderate improvements in discrimination and overall prediction error. The validation AUC increased from 0.716 to 0.744 for logistic regression (Delta AUC = 0.028; paired DeLong *p* = 0.041), from 0.817 to 0.843 for random forest (Delta AUC = 0.026; paired DeLong *p* = 0.058), and from 0.862 to 0.887 for the neural-network model (Delta AUC = 0.025; paired DeLong *p* = 0.047). Brier scores also improved modestly after adding TCM syndrome elements. Decision-curve analysis suggested a small additional net benefit for the extended models across threshold probabilities of approximately 0.25–0.55.

### 3.4 Calibration, clinical utility, and strengthened internal validation

**Figure 3:**
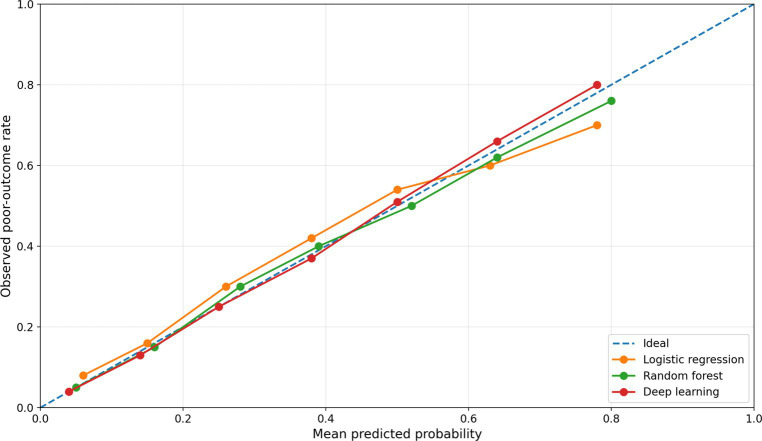
Calibration curves in the hold-out validation cohort.

Calibration results are summarized in Table 4 and Figure 3. In the hold-out validation cohort, the neural network had the lowest Brier score (0.123), with calibration intercept 0.03 and calibration slope 0.94. Random forest showed a Brier score of 0.138, calibration intercept −0.06, and calibration slope 0.86. Logistic regression showed a Brier score of 0.163, calibration intercept −0.02, and calibration slope 0.91.

**Table 4:** Calibration, model comparison, and strengthened internal validation.

Model	Brier score	Calibration intercept	Calibration slope	AUC comparison	Strengthened internal validation
Logistic regression	0.163	−0.02	0.91	Reference	Bootstrap AUC 0.738; repeated CV AUC 0.746; temporal AUC 0.731
Random forest	0.138	−0.06	0.86	vs LR: *p* = 0.032	Bootstrap AUC 0.827; repeated CV AUC 0.835; temporal AUC 0.820
Deep learning	0.123	0.03	0.94	vs LR: *p* = 0.006; vs. RF: *p* = 0.174	Bootstrap AUC 0.862; repeated CV AUC 0.873; temporal AUC 0.861

**Figure 4:**
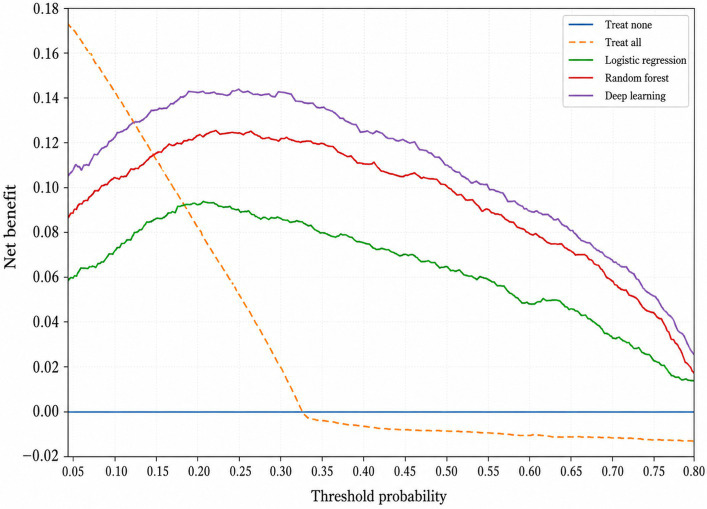
Decision-curve analysis in the hold-out validation cohort.

Decision-curve analysis suggested that the random forest and neural-network models provided greater net benefit than treat-all or treat-none strategies across approximate threshold probabilities of 0.20–0.65, with only modest incremental net benefit of the neural network over random forest (Figure 4). The decision-curve findings should be interpreted as internal evidence of potential clinical utility rather than proof of transportability to other centers.

In bootstrap optimism correction, the optimism-corrected AUCs were 0.738 for logistic regression, 0.827 for random forest, and 0.862 for the neural network. Repeated five-fold cross-validation yielded mean AUCs of 0.746, 0.835, and 0.873, respectively. Temporal validation using earlier years for training and later years for testing yielded AUCs of 0.731, 0.820, and 0.861, respectively.

A direct comparison with established prognostic scores was not performed because the variables required to calculate modified Erasmus GBS Outcome Score or Erasmus GBS Respiratory Insufficiency Score were not consistently available. Consequently, the results should be interpreted as internal comparisons among the newly developed models and a regression baseline, not as evidence of superiority over established scores.

### 3.5 Exploratory analysis of granular functional outcome

Because dichotomizing HFGS at > = 3 may lose information, exploratory sensitivity analyses examined ordinal 12-month HFGS and change in HFGS from admission to 12 months. The predicted neural-network risk correlated moderately with ordinal 12-month HFGS (Spearman rho = 0.56), and the mean absolute error for ordinal HFGS approximation was 0.72 grades. The same major predictors remained influential.

### 3.6 Variable importance

**Figure 5:**
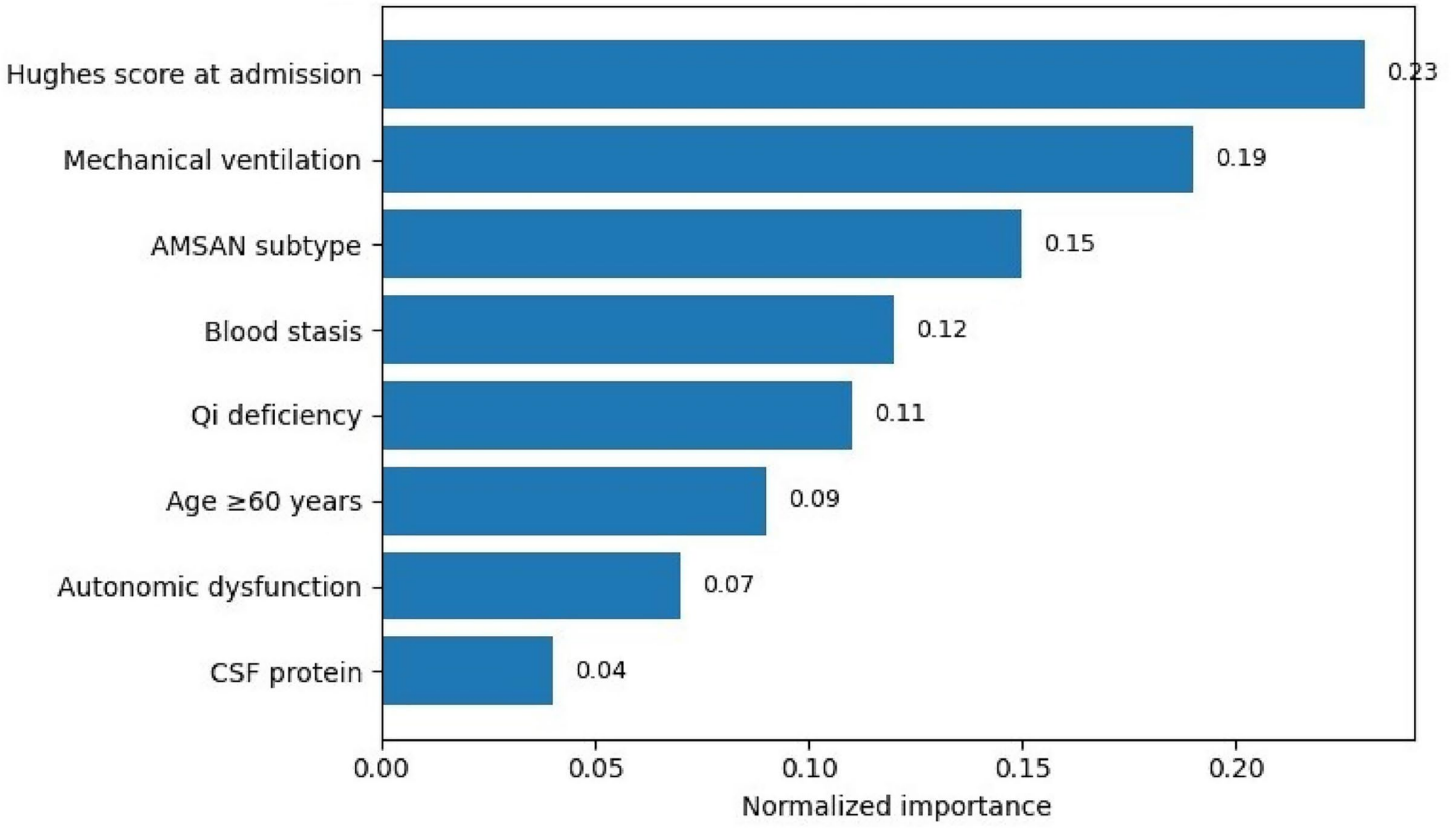
Ranked importance of the leading predictors in the deep learning model. The most influential variables included admission HFGS score, mechanical ventilation, AMSAN subtype, blood stasis, qi deficiency, age > = 60 years, autonomic dysfunction, and cerebrospinal fluid protein.

Feature-importance analysis suggested that the neural network placed greatest weight on admission HFGS score, need for mechanical ventilation, AMSAN subtype, blood stasis, qi deficiency, age > = 60 years, autonomic dysfunction, and cerebrospinal fluid protein (Figure 5). This profile indicates that the model integrated established markers of disease severity with syndrome-level information derived from TCM assessment.

## 4 Discussion

This study developed and internally validated an acute-phase dynamic/discharge-time prognostic framework for GBS that combines conventional clinical variables with TCM syndrome features. The model is not described as an admission-only early prediction tool because several predictors, including nadir HFGS score and mechanical ventilation, are known during acute hospitalization. This clarification reduces the risk of misinterpretation and addresses the possibility of data leakage.

The neural-network model achieved the highest numerical AUC and the lowest Brier score, whereas random forest also performed well. Importantly, the neural network was statistically superior to logistic regression but not to random forest in the internal hold-out validation cohort. Therefore, the manuscript does not claim deep-learning superiority over random forest. The results indicate only that, within this internally validated dataset, nonlinear models produced better numerical performance than the regression baseline, while the apparent advantage of the neural network requires confirmation in larger and external cohorts.

The clinical predictors identified in the present study are consistent with prior work. Mechanical ventilation and admission HFGS score reflect severe acute neurological involvement, and AMSAN reflects axonal injury, which has been associated with poorer recovery in GBS (7, 8, 19). These findings support the clinical plausibility of the model.

The inclusion of TCM syndrome elements is a distinctive aspect of this study. Qi deficiency and blood stasis have been investigated as TCM syndrome constructs in neurological and cardiovascular conditions (26–28). Qi deficiency and blood stasis remained associated with poor 12-month outcome after adjustment and ranked among the more influential variables in the machine-learning model. In the analysis, adding TCM syndrome elements to conventional biomedical predictors was associated with modest improvements in validation AUC, Brier score, and decision-curve net benefit, although the magnitude of improvement was small and should not be overinterpreted. These findings should be interpreted cautiously. The syndrome elements may act as markers of clinically observed vulnerability, disease burden, or recovery reserve, but the present retrospective design cannot establish mechanistic or causal relationships. They should therefore be considered hypothesis-generating and in need of prospective validation.

For practical use, the model could be implemented as a web-based calculator or embedded into the electronic medical record. At discharge, clinicians would enter or automatically import acute-phase variables, electrophysiological subtype, laboratory findings, and standardized TCM syndrome elements. The system would output an individualized probability of poor 12-month functional outcome and a risk category, together with the most influential patient-specific variables. High-risk patients could be prioritized for rehabilitation planning, respiratory and autonomic monitoring, patient counseling, and earlier follow-up appointments. The tool should not replace clinical judgment, and all thresholds should be locally validated before clinical deployment.

This study has several limitations. First, it was retrospective and single-center, so selection bias and limited transportability to other settings cannot be excluded. Second, the validation strategy was internal: the primary assessment used a single stratified random hold-out split, strengthened by bootstrap optimism correction, repeated cross-validation, and temporal validation, but no independent external multicenter cohort was available. A single random split can be unstable, particularly with modest event numbers, and external validation is necessary before clinical implementation. Third, although the sample size is reasonable for a GBS cohort, the number of poor-outcome events is modest for complex neural-network modeling, and overfitting remains possible despite dropout, early stopping, and resampling safeguards. Fourth, item-level missingness was low and handled using training-only imputation procedures, but retrospective missing data may still introduce bias. Fifth, a formal comparison with established prognostic scores such as the modified Erasmus GBS Outcome Score or Erasmus GBS Respiratory Insufficiency Score could not be performed because required score variables were not consistently available; therefore, this study should not be interpreted as showing superiority over existing validated tools. Sixth, some potentially informative predictors, such as immune biomarkers, rehabilitation dose, and serial electrophysiological changes, were not consistently available. Seventh, TCM syndrome differentiation was standardized within the hospital, but external reproducibility requires further evaluation. Future studies should perform prospective, multicenter external validation, collect variables required for established GBS prognostic scores, and assess whether adding TCM variables improves performance beyond both biomedical predictors and validated clinical scores.

## 5 Conclusion

In this retrospective single-center cohort of 525 patients with GBS, acute-phase clinical variables and TCM syndrome elements were associated with poor 12-month functional outcome. Nonlinear machine-learning models showed better numerical performance than logistic regression, and the neural-network model had the highest AUC and lowest Brier score; however, it was not statistically superior to random forest in the internal hold-out validation cohort. No inference of superiority over random forest or established validated prognostic scores should be made. The model should be regarded as an internally validated discharge-time risk-stratification tool requiring external validation, prospective calibration, comparison with established scores when required variables are available, and implementation testing before clinical use.

## Data Availability

The raw data supporting the conclusions of this article will be made available by the authors, without undue reservation.
