# Supplementary material for: Predicting 12-month functional outcome in Guillain-Barré syndrome by combining acute-phase clinical data and traditional Chinese medicine syndrome features: a retrospective machine learning study
Source: Front Neurol. 2026 Jul 29;17:1859051. doi: 10.3389/fneur.2026.1859051 (PMC13461497; doi:10.3389/fneur.2026.1859051)
Supplement: Supplementary file 1 [file Table_1.docx]

# **Supplementary material**

Supplementary Table S1. Complete candidate feature set and preprocessing plan

| **Domain** | **Candidate variables** | **Coding/preprocessing** | **Available by index time** |
| --- | --- | --- | --- |
| Demographics | Age, sex, BMI | Age continuous and >=60 sensitivity; sex binary; BMI continuous standardized | Admission |
| Disease timing | Antecedent infection, time from onset to admission | Binary; continuous standardized | Admission |
| Clinical severity | Cranial nerve involvement, autonomic dysfunction, mechanical ventilation, HFGS at admission, HFGS at nadir | Binary indicators; HFGS continuous/ordinal; standardized when continuous | Admission to acute discharge |
| Laboratory | CSF protein, serum albumin, C-reactive protein | Continuous; imputed and standardized using training data only | During acute hospitalization |
| Electrophysiology | AIDP, AMAN, AMSAN | One-hot encoded; AIDP reference in regression | During acute hospitalization |
| Treatment-related acute-care variables | IVIG, plasma exchange, early rehabilitation referral | Binary indicators; retained for exploratory models only if available before discharge | During acute hospitalization |
| TCM syndrome elements | Qi deficiency, dampness accumulation, blood stasis, phlegm turbidity, yin deficiency, yang deficiency, heat toxin | Each element coded binary; multiple elements allowed | Within 48 hours of admission |

Supplementary Table S2. Additional sensitivity and validation analyses

| **Analysis** | **Purpose** | **Result** |
| --- | --- | --- |
| TCM inter-rater reliability | Standardization of syndrome differentiation | Qi deficiency kappa=0.78; dampness kappa=0.72; blood stasis kappa=0.75; overall kappa=0.74 |
| Ordinal HFGS sensitivity analysis | More granular functional outcome than HFGS >=3 | Neural-network risk vs ordinal 12-month HFGS: Spearman rho=0.56; MAE=0.72 HFGS grades |
| Decision-curve analysis | Clinical utility across risk thresholds | Machine-learning models showed net benefit over treat-all/treat-none at thresholds 0.20-0.65 |
| Temporal validation | Stronger internal validation without external cohort | Deep learning AUC=0.861; random forest AUC=0.820; logistic regression AUC=0.731 |

Supplementary Table S3. Incremental value of adding TCM syndrome elements to conventional biomedical predictors

| **Model class** | **Feature set** | **Validation AUC (95% CI)** | **Brier score** | **Calibration slope** | **Paired comparison** | **Clinical-utility summary** |
| --- | --- | --- | --- | --- | --- | --- |
| Logistic regression | Biomedical predictors only | 0.716 (0.622-0.810) | 0.176 | 0.88 | Reference | Lower net benefit than extended model across thresholds 0.25-0.55 |
| Logistic regression | Biomedical + TCM syndrome elements | 0.744 (0.655-0.833) | 0.163 | 0.91 | Delta AUC=0.028; DeLong P=0.041 | Small added net benefit across thresholds 0.25-0.55 |
| Random forest | Biomedical predictors only | 0.817 (0.747-0.887) | 0.149 | 0.82 | Reference | Lower net benefit than extended model across thresholds 0.25-0.55 |
| Random forest | Biomedical + TCM syndrome elements | 0.843 (0.780-0.906) | 0.138 | 0.86 | Delta AUC=0.026; DeLong P=0.058 | Small added net benefit; borderline AUC comparison |
| Deep learning | Biomedical predictors only | 0.862 (0.799-0.925) | 0.134 | 0.90 | Reference | Lower net benefit than extended model across thresholds 0.25-0.55 |
| Deep learning | Biomedical + TCM syndrome elements | 0.887 (0.831-0.943) | 0.123 | 0.94 | Delta AUC=0.025; DeLong P=0.047 | Small added net benefit across thresholds 0.25-0.55 |

**Supplementary Table S4. Feature-selection process, final model variables, encoded input dimension, and neural-network parameter count**

| **Item** | **Details** |
| --- | --- |
| Candidate feature pool | Demographics, disease timing, acute neurological severity, laboratory values, electrophysiological subtype, acute-care variables, and TCM syndrome elements listed in Supplementary Table S1 |
| Selection method | Training-data selection based on clinical prespecification plus univariable association with poor 12-month outcome at P<0.10 |
| Final biomedical predictors | Age >=60 years; time from symptom onset to admission; autonomic dysfunction; mechanical ventilation; admission HFGS score; nadir HFGS score; cerebrospinal fluid protein; C-reactive protein; electrophysiological subtype |
| Encoding of electrophysiological subtype | Two dummy variables: AMAN and AMSAN; AIDP as reference |
| Final TCM predictors in extended models | Qi deficiency; blood stasis |
| Encoded input dimension | Biomedical-only reference models: 10 encoded inputs; extended models: 12 encoded inputs |
| Final variables by model | Logistic regression, random forest, and neural network used the same selected variables within each biomedical-only or extended feature set |
| Neural-network trainable parameters | Biomedical-only model: 3,329; extended model: 3,457 |

**Supplementary Table S5. Item-level missingness and imputation strategy**

| **Variable** | **Missing values in analytic cohort, n (%)** | **Handling** |
| --- | --- | --- |
| Age | 0 (0.0) | No imputation |
| Sex | 0 (0.0) | No imputation |
| BMI | 15 (2.9) | Imputed if used in sensitivity/candidate analysis |
| Antecedent infection | 0 (0.0) | No imputation |
| Time from symptom onset to admission | 0 (0.0) | No imputation |
| Cranial nerve involvement | 0 (0.0) | No imputation |
| Autonomic dysfunction | 4 (0.8) | Training-only imputation |
| Mechanical ventilation | 0 (0.0) | No imputation |
| Admission HFGS score | 0 (0.0) | No imputation |
| Nadir HFGS score | 3 (0.6) | Training-only imputation |
| Cerebrospinal fluid protein | 30 (5.7) | Training-only imputation |
| Serum albumin | 17 (3.2) | Training-only imputation if used in candidate analysis |
| C-reactive protein | 22 (4.2) | Training-only imputation |
| Electrophysiological subtype | 0 (0.0) in analytic cohort | Unavailable or unclassifiable subtype excluded before cohort splitting |
| Acute immunotherapy | 0 (0.0) | No imputation |
| Early rehabilitation referral | 8 (1.5) | Training-only imputation if used in candidate analysis |
| TCM syndrome elements | 0 (0.0) | No imputation |
| 12-month HFGS outcome | 0 (0.0) in analytic cohort | Missing or unverifiable outcome excluded before cohort splitting |
